# Complete duodenal necrosis associated with non-traumatic duodenal hematoma requiring emergent pancreatico-duodenectomy

**DOI:** 10.1016/j.ijscr.2019.11.026

**Published:** 2019-11-19

**Authors:** Jennifer Koichopolos, Jonathan Keow, Jeremy Parfitt, Cathy Yoshy, Daniele Wiseman, Kenneth Leslie

**Affiliations:** a Department of Surgery, London Health Sciences Center, London, ON, Canada; b Schulich School of Medicine & Dentistry, Western University, London, ON, Canada; c Department of Pathology, London Health Sciences Center, London, ON, Canada; d Department of Radiology, London Health Sciences Center, London, ON, Canada

**Keywords:** Duodenal hematoma, Duodenal necrosis, Pancreatitis, Pancreaticoduodenectomy

## Abstract

**Introduction:**

Duodenal necrosis is a rare complication of acute pancreatitis but can occur given the shared blood supply to the head of the pancreas and the duodenum.

**Presentation of case:**

A 55-year-old male presented with acute-on-chronic pancreatitis and a duodenal hematoma. The hematoma expanded to occlude the biliary tree and, shortly after, the duodenum necrosed and perforated. The patient required an emergent pancreaticoduodenectomy performed in two stages.

**Discussion:**

Surgical management is complex and a difficult challenge for a general surgeon. Many advocate for wide drainage to create a controlled fistula using a malecot through the wall defect/separate duodenotomy/a retrograde jejunostomy tube. This case represents an extreme variation on this issue which was best managed by definitive resection given the extent of the necrosis.

**Conclusion:**

This case report demonstrates that duodenal hematoma and necrosis should be recognized as part of the spectrum of consequences of acute pancreatitis. General surgeons should have a surgical approach to this complication whether that be diversion or definitive resection.

## Highlights

• Duodenal hematoma and necrosis should be recognized as part of the spectrum of consequences of acute pancreatitis.
• Surgical management is complex and challenging.
• General surgeons should have a surgical approach to this complication whether that be diversion or definitive resection.
• Definitive resection should be completed in multiple steps if there is extensive necrosis and the patient is clinically unwell.

## 1 Introduction

Necrosis of the gastrointestinal tract occurs rarely in the setting of acute necrotizing pancreatitis and carries a mortality of 40 % [1]. The most common sites of infarction are the splenic flexure of the transverse colon and the proximal jejunum which is likely due to infarction of the feeding arteries [[2], [3], [4], [5], [6], [7], [8], [9]]. Duodenal necrosis is very uncommon site in this setting however it can occur given the shared blood supply to the head of the pancreas and the duodenum [10]. Other causes of duodenal necrosis are trauma, ingestion of corrosive substances, vasculitis and high jejunal obstruction [10]. There are only a small number of case reports that discuss acute management in this situation [[11], [12], [13]]. This case reviews management of an extensive duodenal hematoma and infarction in the setting of acute-on-chronic pancreatitis. This case is reported in line with the SCARE criteria [14].

## 2 Patient information

**Fig. 1:**
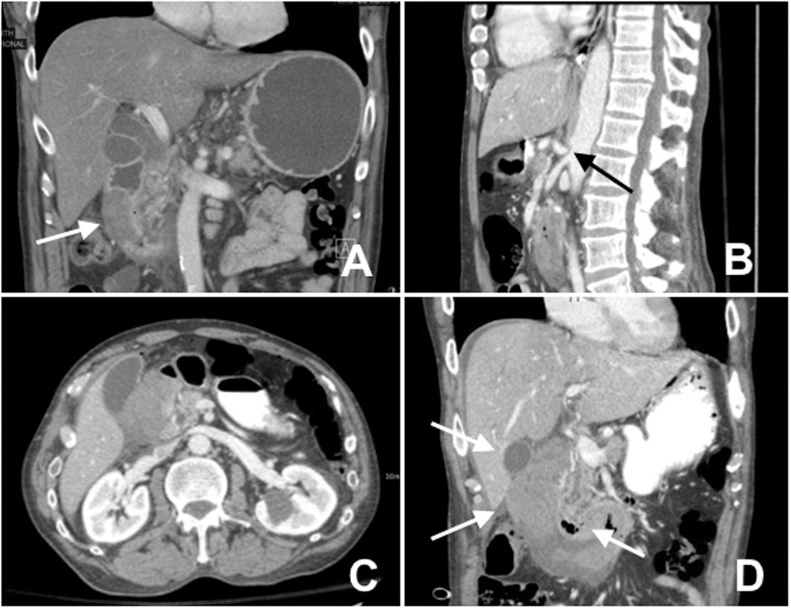
(A) Initial CT scan of patient MM demonstrates a hematoma in the lateral wall of the duodenum. (B) Severe celiac artery stenosis likely from median arcuate ligament. (C) Enlarged pancreaticoduodenal arteries that may have contributed to the formation of the patient’s hematoma. (D) CT performed after patient clinically deteriorated demonstrated a duodenal hematoma extended from D1–D3 with extraluminal gas suggested a perforation.

A 55-year-old male who presented with new onset of abdominal pain to a peripheral hospital. He had a past history of ulcerative colitis, alcoholic pancreatitis, non-alcoholic steatohepatitis, COPD and GERD. His blood work demonstrated pancreatitis (lipase 1440), and jaundice (total bilirubin 98, direct bilirubin 73.9). A CT abdomen/pelvis was performed which showed a new duodenal mass with obstruction (Fig. 1A). In retrospect, the CT additionally demonstrated severe celiac artery stenosis likely from median arcuate ligament (Fig. 1B), which resulted in enlargement of the pancreaticodudenal arteries near the hematoma as a collateral pathway to fill the celiac artery in retrograde from the SMA (Fig. 1C). An esophagogastroduodenoscopy was completed which demonstrated a submucosal mass with yellow to pink hew. He became febrile on day 4 of admission and was placed on piperacillin-tazobactam. The patient was then transferred to a tertiary care center.

On arrival, the patient was afebrile with ongoing jaundice (Total bilirubin 80, direct bilirubin 51). An MR pancreas was performed which suggested that the mass in the duodenum was likely a hematoma.

He required drainage of his biliary tree and an ERCP was attempted for biliary decompression which was unsuccessful. The following morning, he was peritonitic with a rigid abdomen. A repeat CT abdomen and pelvis was performed which demonstrated free air and mild free fluid in keeping with bowel perforation presumably from the duodenum (Fig. 1D). The duodenal hematoma had enlarged into the 3rd part duodenum and duodenal cap. The common bile duct was enlarged and there was mild intrahepatic biliary dilatation-increased since previous exam.

**Fig. 2:**
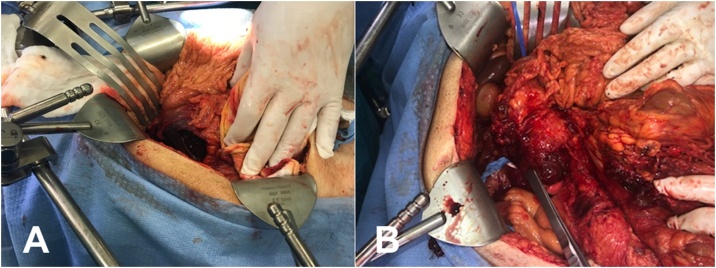
(A) Initial exposure of the duodenum via a kocher maneuver demonstrates a large perforation of the lateral wall. (B) Further dissection demonstrates hematoma dissecting through the duodenal wall from D1–D4.

The patient was taken to the operating room urgently as he was becoming increasingly hemodynamically unwell. He was found to have a completely necrotic duodenum from D1-D4 (Fig. 2A) with the lateral wall widely open and extruding bile (Fig. 2B). The difficult decision to perform an emergent damage-control pancreaticoduodenectomy was made given the extent of the necrosis and size of the defect in the lateral wall. The defect size was a critical factor in this decision as we felt there would be significant difficulty in successfully diverting gastrointestinal contents (ex pyloric exclusion) or making a controlled fistula. The pancreas had signs of chronic pancreatitis during dissection, which was later confirmed on pathology. The resection was performed and the patient was taken the intensive care unit with an open abdomen with planned reconstruction after resuscitation. This 24–48 h between ORs was intended to resuscitate and clinically optimize the patient given his hemodynamic instability in the OR and there was no concern for the perfusion of the residual bowel that was left at the end of the first case and we did not feel a second look laparotomy would be required solely for contamination.

**Fig. 3:**
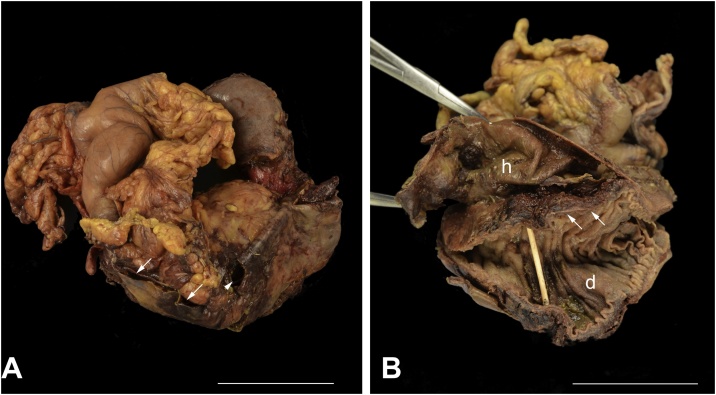
(A) Gross examination of the excised pancreaticoduodenectomy specimen revealed a dusky serosa with perforations (arrowhead), and multiple serosal defects with extrusion of blood clot from the suspected hematoma cavity (arrows). Scale bar = 5 cm. (B) Longitudinal opening of the duodenum (d) along the antimesenteric border reveal a large subserosal hematoma cavity (h) anatomically distinct from the duodenal lumen (arrows). The duodenal lumen is supported by a wooden probe. Scale bar = 5 cm.

**Fig. 4:**
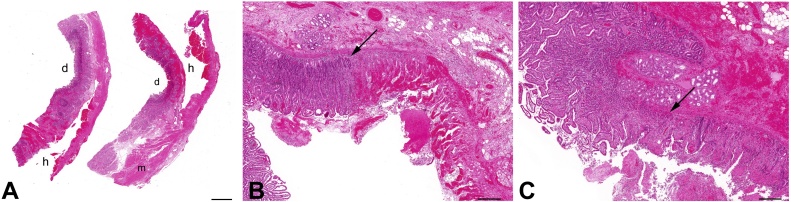
(A) Microscopic examination of representative duodenal sections reveal a large dissecting hematoma cavity (h) originating from within the muscularis propria layer (m), distinct from the duodenal lumen (d). Scale bar = 2 mm. (B) Sections of duodenum demonstrate an abrupt transition (arrow) from healthy duodenal epithelium (left) to an acutely ischemic epithelium (right), marked by hemorrhage, congestion and a neutrophilic infiltrate. Areas of the lamina propria are intensely hyalinized. Scale bar = 0.5 mm. (C) Sections of duodenum demonstrate an abrupt transition (arrow) from healthy duodenal epithelium (left) to an acutely ischemic epithelium (right), marked by epithelial necrosis, loss of the superficial epithelium, and withering crypts. Scale bar = 0.5 mm.

Careful examination of the excised specimen revealed multiple defects within the serosa overlying the duodenum (Fig. 3A, arrowhead). These defects measured between 3 to 5 cm in size, with extensive blood clot extruding from the suspected hematoma cavity (Fig. 3A, arrows). Once the specimen was opened, there were areas of dusky duodenal mucosa and focal necrosis, and there was a clear plane of serosal-mucosal dissection along the majority of the duodenum. This plane of dissection appeared to generate a large 16 cm hematoma cavity (Fig. 3B, h), anatomically distinct from the lumen of the duodenum (Fig. 3B, d). Microscopic examination of the affected areas demonstrated that the hematoma cavity originated from within the muscularis propria layer (Fig. 4A, mp), with areas of abrupt transition to acute ischemic enteritis (Fig. 4B and C, arrows). These were areas with ischemic epithelium and epithelial necrosis, with marked hemorrhage, congestion, and hyalinization of the lamina propria.

**Fig. 5:**
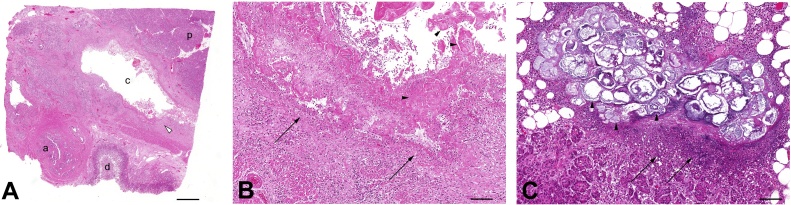
(A) Sections near the ampulla of Vater (a) between the pancreas (p) and duodenum (d) show a cystically dilated duct (c) containing large concretions which are commonly seen in chronic pancreatitis. Scale bar = 1 mm. (B) Examination on higher magnification of these dilated cysts (A, open arrowhead) shows multiple concretions (arrowhead) with an eroded and ulcerated cyst wall (arrows) overlying a neutrophil-rich acute on chronic inflammatory process. Scale bar = 0.2 mm. (C) Sections of excised pancreas demonstrate a patchy areas of saponification and fat necrosis (arrowheads), as well as an intense neutrophilic infiltrate (arrows), in keeping with the classical appearance of acute pancreatitis. Scale bar = 0.2 mm.

Microscopic examination of the proximal pancreatic head revealed several prominent cystically dilated pancreatic ducts (Fig. 5A, c) containing eosinophilic concretions commonly seen in chronic pancreatitis (Fig. 5B, arrowhead). Closer examination of these dilated ducts revealed an eroded and ulcerated wall, with a surrounding acute on chronic inflammatory process (Fig. 5B, arrows). The majority of the submitted pancreatic sections appeared healthy and intact, but there were patchy areas of acute pancreatitis demonstrating an intense neutrophilic infiltrate (Fig. 5C, arrow), fat necrosis and saponification (Fig. 5C, arrowheads). This histologic evidence was consistent with the clinical presentation of both acute and chronic pancreatitis.

Reconstruction was performed on postoperative day 2 in a standard reconstruction with a two-layer pancreatico-jejunostomy (PJ), a single-layer hepatico-jejunostomy and a handsewn double-layer gastrojejunostomy. A total pancreatectomy was not considered as the cut edge of the pancreas was healthy and, despite the higher risk of initial complications by performing a PJ, the benefit of residual pancreas in the long-term out weighted these risks in our opinion. On postoperative day 12 he experienced a fascial dehiscence and required a third operation to washout the abdomen and required placement of retention sutures. Subsequent to this last procedure, he developed an infection which grew E. coli, Raoultella, and mixed flora from the drain in his abdomen. He required IV imipenem and fluconazole. He proceeded to have a type II pancreatojejunostomy leak, which was managed medically with octreotide and TPN. He was repatriated to his home hospital on post-operative day 36 once weaned off of TPN.

## 3 Discussion

Duodenal necrosis is a rare complication of pancreatitis. The underlying pathophysiology is related to vascular compromise and resulting ischemia. Pancreatitis releases enzymes and inflammatory products which can cause thrombosis of the anterior and posterior superior and inferior pancreaticoduodenal arteries or can directly affect the adjacent duodenum. This leads to transmural infarction of the duodenal wall [11,13]. Most commonly the 2^nd^ and 3^rd^ portions of the duodenum are involved as the 1^st^ and 4^th^ portions are supplied by separate arteries (branches of the gastroduodenal and superior mesenteric arteries) [12,13].

Intramural hematoma may be part of this pathologic process and may cause rupture of the duodenal wall causing peritonitis and shock [12]. In this patient, the likely process based on CT findings is that the enlarged pancreaticodudenals placed a large arcade at risk of erosion as a result of pancreatitis possibly causing a hematoma and then pressure necrosis. This is theorized to be part of the same process that can also thrombose vessels: erosion of a duodenal blood vessel by pancreatic autodigestion [12].

Most often, necrosis affects the medial side of the duodenum as seen in previous case reports by Takeyama and Sakorafas [11,13]. Our case unusually involved the lateral wall. This corresponds with the hypothesis that necrosis is primarily due to vascular insult as the distal side of the duodenal loop should be more vulnerable under ischemia [11].

The diagnosis is currently made on axial imaging. However, if the diagnosis is made intra-operatively, bile-stained peritoneal fluid is a sign of duodenal perforation and requires complete inspection of the entire duodenum [13]. If perforation is suspected based on the severity of disease but the location cannot be found, injection of methylene blue through the nasogastric tube may provide a definitive diagnosis [13].

Surgical management is complex and a difficult challenge for a general surgeon. Primary repair is not recommended as the rate of failure is high due to the surrounding inflammation and tissue friability. Other repairs similarly may fail due to these issues such as a onlay-type Roux- en-Y duodenojejunostomy. Other case reports have recommended wide drainage to create a controlled fistula using a malecot through the wall defect/separate duodenotomy/ a retrograde jejunostomy tube. Pyloric exclusion with gastrojejunostomy is an additional option [13].

In this patient’s case, the plan in advance of surgery was a more conservative surgical option. However, given the extent of necrosis and the perforation of almost the entire lateral wall of the second and third portions of the duodenum, a damage-control pancreaticoduodenectomy was performed. There are limited case reports that advocate this approach [12]. We recommend performing this surgery in a damage-control fashion, as in trauma, performing the resection and reconstruction in multiple steps given that these patients are generally unwell and have limited reserve at the time of their perforation.

This case report demonstrates that duodenal hematoma and duodenal necrosis should be recognized as part of the spectrum of rare consequences of pancreatitis. General surgeons should have a surgical approach to this complication whether that be diversion or resection given the high morbidity and mortality of this disease.

## Sources of funding

There is no funding for our work.

## Ethical approval

There is no ethical approval at our institution for case reports.

## Consent

Patient has consented to this case report and there is a sentence in the manuscript that states this.

## Author’s contribution

Jennifer Koichopolos – main author, did case review and literature review and wrote paper.

Jonathan Keow – wrote pathology descriptions and created pathology figures.

Jeremy Parfitt – staff pathologist that reviewed Jonathan Keow’s work.

Daniele Wiseman and Cathy Yoshy – staff radiologist that reviewed the imaging in combination and selected images for the paper.

Ken Leslie – PI for this case report, reviewed the paper in its entirety and suggested changes.

## Registration of research studies

researchregistry4803.

## Guarantor

Dr Ken Leslie is the guarantor of this study.

## Provenance and peer review

Not commissioned, externally peer-reviewed.

## Declaration of Competing Interest

No authors have conflicts, financial or personal relationships with other people or organizations that could influence our work.
